# 간호사의 그릿, 셀프리더십 및 팔로워십이 근거기반실무역량에 미치는 영향: 서술적 상관관계 연구

**DOI:** 10.7586/jkbns.25.016

**Published:** 2025-05-20

**Authors:** Ha-young Kim, Jin-il Kim

**Affiliations:** 1 Jeju National University Hospital, Jeju, Korea; 1 제주대학교병원; 2 Department of Nursing, Health and Nursing Research Institute, Jeju National University, Jeju, Korea; 2 제주대학교 간호학과·건강과간호연구소

**Keywords:** Evidence-based practice, Leadership, Grit, Followership, 근거기반실무역량, 리더십, 그릿, 팔로워십

## Abstract

**Purpose:**

This study examined the associations of grit, self-leadership, and followership with nurses’ competency in evidence-based practice in South Korea.

**Methods:**

This descriptive correlational study included 203 nurses from a general hospital. Data collection was conducted from May 8 to June 21, 2024.

**Results:**

The mean score for competency in evidence-based practice was 4.16 of 7. Competency in evidence-based practice correlated positively with grit (r = .62, *p* < .001), self-leadership (r = .54, *p* < .001), and followership (r = .72, *p* < .001). Followership (β = 0.53, *p* < .001), grit (β = 0.24, *p* < .001) and research experience (β = 0.11, *p* = .024) significantly influenced competency in evidence-based practice. The model’s explanatory power was calculated to be 55.1%.

**Conclusion:**

Followership and grit are key factors in developing strategies to enhance nurses’ competency in evidence-based practice. Additionally, providing support for research experience in clinical settings may further strengthen competency in evidence-based practice.

## 서론

### 1. 연구의 필요성

근거기반실무는 임상에서 가장 관련성이 크고 타당한 근거에 기반하여 의사결정을 하는 것이고, 근거기반실무역량은 다양한 영역에서 지식, 기술 및 태도를 통합하는 것으로 정의되고 있다[1]. 이에 보건의료체계 내에서 적절히 수행되고 기술된 근거기반실무에 더 많이 접근하게 됨에 따라 의료의 질과 결과가 향상될 것으로 기대할 수 있다[2]. 그리고 근거기반실무는 대상자, 병원 및 보건의료체계에 긍정적 영향을 미치는 것으로 보고되고 있고, 여러 의료기관에서 표준으로 여겨지고 있다[3]. 이처럼 근거기반실무에 대한 중요성이 강조되고 있으나 간호사가 인식하고 있는 근거기반실무 지식, 기술, 태도 및 신념은 중간에서 높은 정도이나 근거기반실무의 이행으로 연결되지 않고 있다는 연구보고가 있었다[4]. 이에 간호사의 근거기반실무의 이행을 높이기 위해 근거기반실무역량에 미치는 영향을 파악하는 것이 필요할 것으로 사료된다. 특히 기초간호학에서 근거기반실무의 중요성이 강조되고 있으며, 수많은 기초간호학 연구들이 임상 간호실무의 근거 마련에 기여하고 있음이 보고되고 있다[5]. 무엇보다 임상에서 비판적 사고와 근거에 기반하여 의사결정을 하여야 하는 간호사에게 있어 기초간호학적 지식은 학부과정에서 함양되어야 할 뿐 아니라 새로운 지식을 적용할 수 있는 역량이 요구된다는 점을 비추어 볼 때[6] 근거기반실무의 이행을 통해 기초간호학 연구 및 교육 발전에 기여할 수 있을 것으로 보인다.

근거기반실무의 속성을 살펴보면 실무에서 의사결정을 내릴 때 최상의 근거를 성실하고, 명확하며 신중하게 이용하는 것으로 설명되고 있다[7]. 이처럼 근거기반실무를 위해서는 성실성이 강조된다는 측면을 살펴볼 때 장기목표를 위한 끈기와 열정을 의미하는 그릿(grit)과의 관계를 파악해 볼 필요성이 있다. 그릿은 다양한 개별 성과영역에서 높은 성취를 예측하는 요인으로 여겨지고 있고, 간호에서도 성공적인 업무수행에 그 중요성이 강조되고 있다[8,9]. 그릿의 정도가 높은 치료사들은 환자에게 개방적, 자발적이며 근거기반실무 이행이 높은 것으로 나타났다[10]. 특히 그릿은 간호사가 변화하는 간호현장에서 전문성을 발휘하기 위해 노력을 지속하게 하는 개념으로 설명되고 있다[11]. 간호사의 그릿 정도는 간호대학생과 유사하거나 낮은 수준으로 나타났고, 이는 변화하는 의료상황으로 인한 스트레스, 압박감 및 소진 등과 관련된 것으로 설명되고 있었다[12].

한편 근거기반실무에서 리더십의 중요성이 강조되고 있고, 보건의료 분야에서도 근거기반실무의 주요 결정인자로서 리더십에 대한 많은 연구가 수행되어 왔다[13]. 다양한 리더십 중 자기 스스로에게 영향을 미치는 과정으로, 자연스럽게 동기부여가 되는 업무 뿐 아니라 동기부여가 되지 않는 업무를 수행하도록 하는 포괄적으로 자기 영향(self-influence) 관점을 의미하는 셀프리더십[14]이 업무성과 및 근거기반실무와 관계가 있음이 보고되고 있다. 셀프리더십은 근로자의 업무몰입과 규범적 몰입 및 업무성과에 긍정적 영향요인으로 보고되고 있다[15]. 또한 권역외상센터 간호사를 대상으로 한 연구에서 셀프리더십과 근거기반실무역량은 정적 상관관계를 나타내는 것으로 나타났다[16]. 셀프리더십은 간호사의 직무와 관련된 만족 및 조직몰입 향상 뿐 아니라 직무 스트레스 감소에도 영향을 주는 요인으로 간호사에게 필요한 역량으로 인식되고 있고, 간호사의 업무성과 향상에 기여할 수 있다[17]. 이러한 점을 고려할 때 셀프리더십이 다양한 임상현장에서 의사결정을 하여야 하는 간호사에게 필요한 근거기반실무역량에 미치는 영향을 파악하는 것이 필요할 것으로 보인다.

팔로워십은 효과적으로 지시에 따르고 리더의 노력을 지원하여 구조화된 조직을 최대한 활용할 수 있도록 하는 능력을 의미한다[18]. 그간 리더십은 간호사의 전문성과 업무성과 향상을 위해 필요한 중요한 역량 중 하나로 인식되고 있다[19]. 그러나 조직의 성공에 기여하는 정도는 리더보다 주로 팔로워가 훨씬 큰 것으로 제시되고 있다[20]. 적극적 팔로워는 업무에 적극적으로 참여하고 양질의 업무성과를 도출할 수 있으며 높은 조직성과 달성에 기여할 수 있는 반면 권위를 추종하는 수동적 팔로워는 리더의 비윤리적인 지시를 받아들일 수 있는 것으로 보고되고 있다[21,22]. 팔로워십은 팔로워십에 대한 간호사의 인식을 조사한 연구에서 팔로워십 정도가 높은 경우 적극적으로 팀 업무에 참여하게 되고 문제해결책을 제공할 수 있는 것으로 나타났다. 또한 간호사는 리더의 역할이해 및 리더의 근거기반 지시 활용을 통해 리더를 더 따르게 되고, 리더가 명확한 의사소통을 할 때 간호사의 업무 참여에 대한 의지가 촉진되는 것으로 보고되었다[23]. 임상에서 근무하는 간호사는 병원의 정책 및 책임자의 계획을 따르는 팔로워지만 최상의 간호를 제공하기 위한 다학제간 팀을 주도하거나 근거기반실무를 주도하기도 한다[24]. 따라서 팔로워십과 근거기반 실무역량 간 관계를 파악하는 것은 근거기반실무 향상을 위해 필요할 것으로 보인다.

그릿, 셀프리더십 및 팔로워십과 근거기반실무역량과의 관계를 파악한 연구를 살펴보면 관련된 연구가 적은 실정이다. 물론 간호사를 대상으로 한 선행연구 중 그릿과 근거기반실무역량 간 상관관계가 있었다는 연구결과[25]와 그릿이 근거기반실무역량에 유의한 영향요인이었다는 보고[26]가 있었고, 셀프리더십과 근거기반실무역량간 상관관계가 있었음을 보고한 연구[16]가 있었다. 그러나 그릿, 셀프리더십 및 팔로워십이 근거기반실무역량에 미치는 영향을 본 연구는 부족한 것으로 나타났고, 특히 팔로워십과 근거기반실무역량 간 관계를 파악한 연구는 거의 찾아보기 어려웠다.

보건의료의 질 향상에 근거기반실무의 수행이 강조되고 있는 시점에서 근거기반실무역량과 관련성이 예상되는 변인을 규명하는 연구가 중요할 것으로 보인다. 이에 본 연구에서는 간호사를 대상으로 그릿, 셀프리더십 및 팔로워십을 변인으로 하여 근거기반실무역량에 미치는 영향을 파악함으로써 간호사의 근거기반실무역량 강화를 위한 전략마련에 기반자료를 제공하고자 한다.

### 2. 연구의 목적

종합병원에 근무하는 간호사를 대상으로 근거기반실무역량에 미치는 영향을 파악하기 위하여 수행되었고, 구체적인 목적은 아래와 같다.

1) 대상자의 일반적 특성, 근거기반실무역량, 그릿, 셀프리더십 및 팔로워십 정도를 분석한다.

2) 일반적 특성에 따른 근거기반실무역량, 그릿, 셀프리더십 및 팔로워십의 차이를 확인한다.

3) 근거기반실무역량, 그릿, 셀프리더십 및 팔로워십 사이의 상관관계를 파악한다.

4) 근거기반실무역량에 미치는 영향요인을 분석한다.

## 연구 방법

### 1. 연구 설계

종합병원에 근무하는 간호사의 근거기반실무역량에 미치는 영향요인을 조사하기 위해 수행된 서술적 상관관계 연구이다.

### 2. 연구 대상

제주대학교병원에 근무하는 1년 이상의 임상경력을 가진 간호사이다. 대상자 수 산정을 위해 G*Power (version 3.1.9.7, Heinrich-Heine-Universität Düsseldorf, Düsseldorf, Germany) [27]을 이용하였고, 예측변수 13개, 중간 효과크기 .15, 검정력 .95, 유의수준 .05로 설정하여 적용한 결과 189명이 산출되었다. 탈락률을 약 15%로 고려하여 222명을 대상으로 설문을 수행하였고, 불성실하게 응답된 설문지 19부가 제외되어 최종 203명이었다.

### 3. 연구 도구

일반적 특성, 그릿, 셀프리더십, 팔로워십 및 근거기반실무역량을 측정하기 위해 구조화된 설문지를 이용하여 자료를 수집하였다. 각 도구 사용을 위하여 원저자 또는 출판사 및 수정ㆍ번안한 연구자의 동의를 받았다.

#### 1) 일반적 특성

일반적 특성은 연령, 성별, 교육수준, 근무경력, 근무형태, 근무장소, 연구관련 교육 유무, 학회가입 및 참여 유무, 연구경험 유무로 구성된 문항으로 측정하였다.

#### 2) 그릿

그릿은 그릿 측정 도구인 Original Grit Scale [8]을 기초로 임상간호사를 대상으로 그릿을 측정하기 위해 개발한 Clinical Nurses Grit Scale [28]으로 측정하였다. 본 도구는 간호 전문가가 되기 위한 열정 5문항, 장기적인 목표달성을 위한 끈기 5문항, 환자 지향성 내적동기 4문항으로 구성되어 있다. ‘항상 그렇지 않다’부터 ‘항상 그렇다’까지 1점에서 4점으로 구성된 Likert 척도로 점수가 높을수록 그릿의 정도가 높은 것을 의미한다. 본 도구의 Cronbach’s α는 Park 등[28]의 연구에서 .91이었고 본 연구에서는 .86이었다.

#### 3) 셀프리더십

셀프리더십은 Abbreviated Self-Leadership Questionnaire (ASLQ) [29]를 한국어로 번안한 셀프리더십 측정도구[30]로 측정하였다. 본 도구는 행동지각 및 의지, 과업동기, 건설적 인지 각각 3문항씩 총 9문항으로 구성되어 있다. '전혀 그렇지 않다'부터 '매우 그렇다'까지 1점에서 5점의 Likert 척도로 점수가 높을수록 셀프리더십의 정도가 높은 것을 의미한다. 본 도구의 Cronbach's α는 Choi [30]의 연구에서는 .87이었고, 본 연구에서는 .90이었다.

#### 4) 팔로워십

팔로워십은 Followership Questionnaire [20]를 한국어로 번안한 도구[31]로 측정하였다. 본 도구는 적극적 참여 10문항과 독립적ㆍ비판적 사고 10문항으로 구성되어 있다. '전혀 그렇지 않다'부터 '매우 그렇다'까지 1점에서 5점의 Likert 척도로 점수가 높을수록 팔로워십의 정도가 높은 것을 의미한다. 본 도구의 Cronbach's α는 Kim [31]의 연구에서는 .89 ~ .95 이었고, 본 연구에서는 .93이었다.

#### 5) 근거기반실무역량

근거기반실무역량은 간호사의 근거기반실무역량을 측정하기 위해 개발된 Evidence-based Practice Questionnaire [32]를 한국어로 번안한 도구[33]로 측정하였다. 본 도구는 근거기반실무에 대한 지식/기술 14문항, 수행 6문항, 태도 4문항의 총 24문항으로 구성되어 있다. '전혀 그렇지 않다'부터 '매우 그렇다'까지 1점에서 7점의 Likert 척도로 점수가 높을수록 근거기반실무역량이 높은 것을 의미한다. 본 도구의 Cronbach's α는 Lim 등[33]의 연구에서는 .93이었고, 본 연구에서는 .93이었다.

### 4. 자료 수집

제주대학교병원의 간호사를 대상으로 자기보고식 설문 방법으로 2024년 5월 8일에서 6월 21일까지 진행하였다. 연구자가 각 부서를 방문하여 부서 관계자에게 연구목적에 대해 설명한 후 부서 관계자를 통해 설명서, 동의서와 설문지를 배부하였다. 연구 대상자에게 연구목적 외에 사용되지 않으며, 기밀보장에 대해 설명서를 통해 설명하여 자발적으로 동의한 간호사에 한하여 서면 동의서를 받고 진행하였다. 설문지는 구조화된 자기보고식 설문으로 구성되었고, 대상자가 작성 후 밀봉할 수 있도록 봉투를 제공하였다. 작성된 설문지는 연구자가 직접 회수하였다.

### 5. 자료 분석

통계 분석을 위해 SPSS version 22.0 (IBM, Armonk, NY, USA)을 이용하였다. 대상자의 일반적 특성, 근거기반실무역량, 그릿, 셀프리더십 및 팔로워십은 실수, 백분율, 평균 및 표준편차로 분석하였다. 대상자의 일반적 특성에 따른 제 변수의 차이 검정은 independent t-test 또는 one-way ANOVA로 분석하였고, Scheffé test로 사후검정을 하였다. 일반적 특성에 따른 제 변수 값의 차이 분석 시 집단 별 30명 미만인 경우는 Shapiro-Wilk test를 이용하여 정규성 검정을 하였고, 정규분포하지 않는 경우 Mann-Whitney U test 또는 Kruskal-Wallis test로 분석하였다. 대상자의 근거기반실무역량, 그릿, 셀프리더십 및 팔로워십 간의 상관관계 분석을 위해 Pearson correlation coefficient를 이용하였다. 대상자의 근거기반실무역량 영향요인은 step-wise multiple regression을 이용하여 분석하였다.

### 6. 윤리적 고려

본 연구는 제주대학교의 생명윤리심의위원회의 승인(승인번호: JJNU-IRB-2024-030-001) 후 수행하였다. 연구 수행 전 대상자에게 연구목적 및 방법 등에 대한 설명문을 제공하여 자발적 연구 참여와 원할 경우 중도에 연구 참여를 거부할 수 있음에 대하여 설명하였다. 또한 대상자의 권익보호, 비밀 보장, 중도 철회에 따른 불이익이 없다는 것과 목적 이외 이용하지 않을 것 및 연구 종료 3년 후 폐기됨을 설명하였다. 소정의 답례품을 자료수집 후 연구대상자에게 제공하였다.

## 연구 결과

### 1. 일반적 특성

평균 연령은 31.3세였으며 30대가 46.8%로 가장 많았고, 여성이 93.6%로 대부분을 차지하였다. 교육수준은 학사 이상의 간호사가 82.3%로 가장 많았다. 대상자의 평균 근무경력은 8.8년이었으며 5년 이상 10년 미만 근무가 39.9%로 가장 많았고, 근무형태는 교대직이 69%로 가장 많았다. 근무장소는 일반병동 41.9%, 중환자실 18.7%, 응급실 13.3% 순이었다. 연구 관련 교육을 받은 경험이 있는 대상자는 66.5%였고, 학회 참석 경험이 있는 대상자는 41.9%였으며 학회에 가입이 되어 있는 대상자는 5.9%로 나타났다. 또한 연구경험이 있는 대상자는 53.2%이었다(Table 1).

### 2. 근거기반실무역량, 그릿, 셀프리더십 및 팔로워십

대상자의 근거기반실무역량은 평균 4.16점/7점이었고, 하위 영역별로는 수행이 4.49점으로 가장 높았고, 태도는 4.28점 및 지식/기술은 3.99점 순으로 나타났다. 그릿은 2.91점/4점이었고, 하위 영역별로 점수가 가장 높은 영역은 3.09점으로 ‘간호 전문가가 되기 위한 열정’이었고, 다음은 ‘환자 지향성 내적동기’ 및 ‘장기적인 목표달성을 위한 끈기’ 순이었다. 셀프리더십은 3.21점/5점이었고, 하위 영역별 점수는 ‘행동지각 및 의지’, ‘건설적 인지’ 및 ‘과제동기’ 순으로 나타났다. 팔로워십은 2.97점/5점이었으며 하위 영역별로는 ‘적극적 참여’가 3.10점, ‘독립적ㆍ비판적 사고’가 2.84점이었다(Table 2).

### 3. 일반적 특성에 따른 근거기반실무역량, 그릿, 셀프리더십 및 팔로워십의 차이

대상자의 근거기반실무역량 차이를 일반적 특성에 따라 비교한 결과 연령(F = 4.60, *p* = .011), 근무경력(χ^2^ = 7.94, *p* = .047), 근무형태(t = 2.46, *p* = .015), 학회가입 및 참여 유무(F = 7.19, *p* < .001), 연구경험 유무(t = 3.57, *p* < .001)에 따라 차이가 있었다. 상근직 간호사에 비해 교대근무 간호사가 그리고 연구경험이 있는 간호사가 없는 간호사에 비해 근거기반실무역량 점수가 높았다. 사후검정 결과 연령이 높은 군에서 낮은 군 순으로, 학회가입 및 참여하는 군에서 학회만 참석한 군, 학회가입 및 참여하지 않은 군 순으로 근거기반실무역량이 높았다.

그릿은 연령(F = 5.65, *p* = .004), 학회가입 및 참여 유무(χ^2^ = 33.63, *p* < .001), 연구경험 유무(t = 2.34, *p* = .020)에 따라 차이가 있는 것으로 나타났다. 또한 연구경험이 있는 간호사가 없는 간호사에 비해 그릿이 높았고, 사후검정 결과 40대 이상이 다른 군보다 높았다. 학회가입 및 참여하는 군이 학회만 가입한 군에 비해 그릿이 높은 것으로 나타났다.

셀프리더십의 경우 연령(F = 4.76, *p* = .010), 학회가입 및 참여 유무(χ^2^ = 11.28, *p* = .010), 연구경험 유무(t = 2.25, *p* = .026)에 따라 차이를 보였다. 연구경험이 있는 군이 없는 군에 비해 셀프리더십 점수가 높았고, 사후검정 결과 40대 이상이 30대 이상보다 비해 셀프리더십 점수가 높은 것으로 나타났다. 학회가입 및 참여하는 군에서 학회만 가입한 군, 학회만 참석한 군 순으로 셀프리더십이 높았다.

팔로워십은 연령(F = 3.89, *p* = .022), 학회가입 및 참여 유무(F = 20.75, *p* < .001), 연구경험 유무(t = 2.60, *p* = .010)에 따라 유의한 차이를 보였다. 사후검정 결과 연구경험이 있는 군이 없는 군에 비해 팔로워십 점수가 높았다. 사후검정 결과 40대 이상이 30대 이상보다, 학회가입 및 참여하는 군이 학회만 가입한 군 보다 팔로워십이 높았다(Table 3).

### 4. 근거기반실무역량, 그릿, 셀프리더십, 팔로워십 간의 상관관계

근거기반실무역량과 그릿, 셀프리더십 및 팔로워십 간의 상관관계를 분석한 결과 모든 변수 간 유의한 상관관계를 보였다. 근거기반실무역량은 그릿(r = .62, *p* < .001), 셀프리더십(r = .54, *p* < .001), 팔로워십(r = .72, *p* < .001)과 유의한 상관관계가 있었다(Table 4).

### 5. 근거기반실무역량의 영향요인

그릿, 셀프리더십, 팔로워십 및 일반적 특성에 따라 근거기반실무역량의 차이가 있었던 연령, 근무경력, 근무형태, 학회가입 및 참여 유무, 연구경험 유무를 투입하여 단계적 회귀분석을 수행하였다. 일반적 특성 중 명목변수에 해당하는 변수는 더미변수로 처리하여 분석하였다. 공차한계는 0.54에서 0.96으로 0.1 이상이었고, 분산팽창인자는 1.04에서 1.85로 기준값인 10보다 작아 다중공선성 문제는 없는 것으로 나타났다.

회귀분석 결과 예측회귀모형은 유의(F = 83.63, *p* < .001)하였으며 팔로워십(β = 0.53, *p* < .001), 그릿(β = 0.24, *p* < .001) 및 연구경험(β = 0.11, *p* = .024)이 근거기반실무역량에 영향을 주는 것으로 나타났다. 변수들의 근거기반실무역량에 대한 설명력은 55.1%이었다(Table 5).

## 논의

본 연구는 종합병원에 근무하는 간호사를 대상으로 근거기반실무역량에 미치는 영향을 규명하여 향후 간호사의 근거기반실무역량 강화에 필요한 기초자료를 제공하고자 수행되었다.

본 연구결과 대상자의 근거기반실무역량은 4.16점/7점으로 나타났다. 이러한 결과는 선행연구와 본 연구가 수행된 병원규모, 소재지역 등이 상이하여 직접 비교에는 무리가 있으나 중소병원 간호사를 대상으로 한 연구에서 나온 4.18점[34]과 유사한 수준이었으나 서울 소재 대학병원에 근무하는 간호사를 대상으로 한 연구에서 나타난 점수인 4.67점[35]보다 낮은 것으로 나타났다. 근거기반실무역량에 미치는 인구사회학적 요인은 다양할 수 있으나 교육수준에 따라 차이가 있으며 특히 석사과정 이상에서 유의하게 높았음을 보고한 선행연구 결과[33]를 토대로 부분적으로 설명될 수 있다. 본 연구 참여자 중 석사 이상의 대상자는 거의 없었기 때문에 이러한 결과가 나왔을 것으로 생각되며 대학원 진학을 장려하거나 연구결과를 임상에 접목시키는 것을 권장하는 등 근거기반실무 확산을 위한 추가적인 노력이 필요할 것으로 보인다. 특히 본 연구에서 지식/기술 영역의 점수가 가장 낮았고, 설문문항 중 수행 영역에서 ‘실무에서 가졌던 의문과 관련된 근거를 찾음’ 문항의 점수는 가장 높았으나 지식/기술 영역에서 ‘실무에서 필요한 지식을 연구 문제로 전환하는 능력 있음’ 문항의 점수는 가장 낮았던 점은 근거기반실무 확산과 관련하여 수행 능력은 향상되었으나 임상에서 확인된 문제를 연구로 전환하는 능력을 보완할 필요가 있다는 주장[36]을 뒷받침해 준다. 따라서 간호사는 간호현장 실무에서 부딪히게 되는 문제와 의문에 대하여 스스로 근거를 탐색할 뿐 아니라 동료와 공유하며 함께 탐색하고 연구문제로 전환하여 해결하는 노력하는 것이 필요할 것으로 보인다.

본 연구결과 연령이 높은 군이 낮은 군에 비하여 근거기반실무역량이 높은 것으로 나타났다. 이러한 결과는 연령이 근거기반실무역량의 유의한 영향요인으로 나타났던 결과[33] 및 연령이 높을수록 근거기반실무역량이 높게 나타난 연구결과[36]와 유사한 결과였다. 이러한 결과는 연령이 높아짐에 따라 다양한 임상경험을 통해 문제 판단능력과 해결능력이 높아질 수 있고[35], 그간 의료기관 인증평가를 통해 근거기반실무의 중요성이 커졌고, 근거기반실무와 관련된 다양한 활동들이 이루어져 왔던 점이 근거기반실무역량에 영향을 미쳤을 것이라는 선행연구 보고[36]를 토대로 연령이 높은 군에서 근거기반실무역량이 높았을 것으로 해석할 수 있다. 따라서 근거기반실무 역량 향상을 위해 간호사의 근무를 지속할 수 있는 임상환경을 마련하고 경력에 따른 프로그램 마련이 필요할 것으로 보인다.

근거기반실무역량에 영향을 주는 요인을 살펴본 결과 가장 큰 영향을 주는 요인은 팔로워십으로 나타났다. 팔로워십이 근거기반실무역량에 미치는 영향을 조사한 연구는 찾아보기 어려웠으나 팔로워로서 간호사는 업무 분담, 경험과 근거에 의해 지지되는 지식, 의사소통을 통해 팔로워십에 참여하게 된다. 이는 안전하고 유능한 그리고 근거기반의 간호실무에 대한 신념을 강화시킨다[23]. 또한 팔로워십은 전문가로 숙달되기 위해 반드시 필요한 것으로 팔로워십에 대한 이해를 통해 간호의 질과 근무환경 및 생산성을 증진시킬 수 있다[37]. 따라서 팔로워십은 근거기반실무에 영향을 미치는 변인으로 생각된다. 간호사의 팔로워십에 대한 유형을 규명한 선행연구에 따르면 간호사의 대부분에 해당하는 팔로워십 유형으로 조직의 목표달성을 위해 아이디어를 적극적으로 제안하고 타인의 의견을 존중하며 리더를 지지하는 발전지향적 팔로워 유형으로 나타났다. 또한 이러한 유형에 속한 간호사는 근무경력이 많은 간호사였으며 풍부한 임상경험을 통해 자발적으로 업무를 수행하기 때문으로 설명하고 있었다[38]. 본 연구에서도 연령이 높을수록 팔로워십 점수가 높았던 것도 선행연구 결과를 지지해준다. 이처럼 풍부한 임상경험을 통해 팔로워십이 개발될 수 있음이 제시되고 있으나 그러나 경력이 낮은 간호사의 팔로워십 향상에 대한 고려도 필요할 것으로 보인다. 팔로워십은 임상경력이 증가에 따라서만 증가될 수 있는 것이 아니라 중재를 통하여 개발될 수 있음을 제시하는 연구가 있었다. 대학생을 대상으로 게임화된 어플리케이션을 이용하여 팔로워십 자기기대(self-expectation)에 미치는 효과를 규명한 연구를 살펴보면 자기실현적 예언(self-fulfilling prophecy) 과정을 촉발함으로써 스스로 좋은 팔로워라고 생각하는 팔로워십 자기기대를 향상시키고, 자기효능감과 개인적 숙련을 높여 긍정적인 팔로워십 사고방식을 강화할 수 있는 것으로 나타났다[39]. 이처럼 단기간에 팔로워십을 증진시킬 수 있었던 선행연구 결과는 경력이 낮은 간호사를 위한 팔로워십 훈련 프로그램 개발에 기초자료가 될 수 있을 것으로 사료된다.

다음으로 근거기반실무역량에 영향을 미치는 요인은 그릿이었다. 이러한 결과는 두 변수간 유의한 상관관계가 있었던 선행연구 결과[25]와 그릿이 근거기반실무역량에 유의한 영향요인이었다는 선행연구결과[26]와 유사한 결과였다. 그릿은 성실성과 관련이 있으며 특히 그릿의 하위 요인인 노력지속은 흥미유지보다 더 강한 상관관계가 있는 것으로 보고되고 있다[40]. 이는 근거기반실무는 최상의 근거를 성실하게 이용하는 것임[7]을 고려할 때 그릿이 근거기반실무역량에 유의한 영향을 미치는 요인으로 볼 수 있다. 또한 약물남용장애 치료사를 대상으로 그릿이 근거기반실무 이행에 미치는 영향을 살펴본 연구에서 그릿이 높은 치료사가 환자에게 더 개방적이고 근거기반실무를 더 잘 이행할 수 있는 것으로 나타났다. 이는 그릿이 높을수록 자신의 경험보다 과학적 근거에 더 가치를 두고 강한 치료동맹(therapeutic alliance)을 통해 근거기반실무 이행을 촉진하기 때문으로 보고되고 있다[10]. 간호사의 근거기반실무의 경험을 토대로 근거기반실무의 장애요인을 조사한 선행연구에 따르면 ‘번거로움’, ‘힘들 것 같음’, ‘복잡하게 생각함’과 같이 부담감을 근거기반실무의 장애요인으로 인식하고 있었다[41]. 임상현장에서 근거기반실무가 번거로운 업무로 인식되고 있음을 고려할 때 장기목표를 위한 끈기와 열정을 의미하는 그릿은 간호사가 근거기반실무를 성공적으로 수행하기 위해 개발해야 할 능력으로 사료된다. 한편, 그릿은 연령이 높아짐에 따라 조직에서의 책임감 증가와 함께 성장되는 것으로 알려져 있다[9]. 이는 본 연구에서 연령이 높을수록 그릿 점수가 높았던 결과를 뒷받침해 준다. 그릿이 높은 간호사는 최상의 근거를 간호에 적용하고 그릿이 높은 연구자는 실무에 필요한 기초 지식을 향상시킬 수 있을 뿐 아니라[42] 그릿은 간호업무성과에 영향을 미치는 요인이므로[9] 근거기반실무역량 향상과 더불어 다양한 임상결과의 향상을 위해 간호사의 그릿의 향상이 필요할 것으로 보인다. 간호사의 그릿을 향상시키기 위한 중재연구는 찾아보기 어려웠으나 청소년을 대상으로 스토리, 초이스맵, 또래교수(peer tutoring), 자기평가 및 언어적 자기교시(verbal self-instruction)로 구성된 프로그램을 적용한 연구가 있었다. 해당 프로그램은 그룹기반의 장기목표에 대한 노력과 관심을 지속시키기 위한 능력 증진에 초점을 맞춘 프로그램으로 9회기의 중재 결과 그릿이 향상된 것으로 나타났다[43]. 따라서 선행연구를 참고하여 간호사들이 임상현장에서 장기목표를 위한 끈기와 열정을 지속하도록 하는 프로그램의 개발 및 운영도 필요할 것으로 보인다.

마지막으로 근거기반실무역량에 영향을 미치는 요인은 연구경험으로 나타났다. 이는 상급종합병원 간호사를 대상으로 근거기반실무역량에 미치는 영향을 규명하였던 연구에서 연구경험이 근거기반실무역량에 유의한 영향요인으로 나온 결과와 일치하였다[44]. 또한 의사를 대상으로 연구훈련 프로그램의 효과를 조사한 연구에서 연구훈련을 통해 연구지식과 연구능력이 향상된 것으로 나타났을 뿐 아니라 관리자와 의사 모두 근거기반실무가 향상되었다고 인식한 것으로 나타났던 결과[45]도 본 연구결과를 뒷받침해 준다. 또한 학회 참석을 통해 다양한 최신 연구 동향을 살필 수 있음을 고려할 때 학회에 참석한다고 응답한 간호사가 그렇지 않은 간호사에 비해 근거기반실무역량이 더 높았던 것도 이러한 해석을 지지해준다. 전문가간 근거기반실무 모형(interprofessional model of evidence-based practice)에 따르면 최상의 근거는 다양한 보건의료 전문가간의 공유된 의사결정의 구성요소로 제시되고 있다. 또한 근거기반실무는 수준 높은 과학적 연구에서 얻은 근거를 실무로 중개하여 최상의 의사결정을 하도록 한다[46]. 그러나 선행연구에 따르면 근거기반실무의 중요성과 다양한 임상결과에 긍정적 영향을 끼침에도 불구하고 연구결과를 임상실무에 적용하는 연구활용과 근거기반실무 간 격차가 존재함을 보고하고 있었다[47]. 그리고 간호사의 전문직 활동을 조사한 국내연구에서도 교육자료개발이나 질향상 활동에 비해 연구활동이나 근거기반간호 활동에 참여한 비율이 적었던 것으로 나타났다[48]. 따라서 간호사의 연구참여를 향상시키기 위해 대학원 진학을 장려하거나 병원 차원의 간호 연구활동 지원이 이루어진다면 간호사의 근거기반실무역량 향상에 도움이 될 것으로 기대된다.

셀프리더십은 근거기반실무역량의 영향요인으로 나타나지 않았다. ASLQ는 셀프리더십의 하위차원 중 자기 단서와 자연적 보상을 측정하는 문항이 축약된 제한점이 있는 것으로 알려져 있다[29]. 이 중 자기 단서는 행동을 직접 변화시키기보다 행동 환경에 더 초점을 두는 간접적 전략이긴 하나[29] 장기간이고 지속적인 습관을 만드는데 중요한 요인으로 단서화가 제시되고 있다. 해야할 일을 떠오르게 하는 단서와 이에 대한 보상을 통해 습관을 형성할 수 있다고 하였다[49]. 근거기반실무는 근거기반실무 유도 습관 형성을 통해 개발될 수 있으므로[50] 이러한 차원을 측정하지 못하였다는 점이 셀프리더십이 근거기반실무역량의 영향요인이 아니었던 것과 연관될 수 있을 것으로 본다.

본 연구의 제한점으로는 일개 지역 일개 종합병원 간호사를 대상으로 수행되었기 때문에 일반화에 제한이 있을 수 있다는 점을 들 수 있다. 또한 연구참여자 중 수간호사와 석사 이상의 간호사가 거의 포함되지 않아 직위와 교육수준에 따른 분석이 어려웠다. 따라서 후속연구를 통해 다양한 규모의 병원 간호사를 대상으로 직위와 교육수준의 범위를 넓혀 추가적인 영향요인을 규명하는 것이 필요하겠다. 그러나 간호사의 근거기반실무역량 향상의 필요성이 증대되고 있는 시점에서 그릿, 셀프리더십 및 팔로워십을 중심으로 간호사의 근거기반실무역량에 미치는 영향을 분석하였다는 점은 간호사의 근거기반실무역량 향상을 위한 전략마련에 기초자료를 제공할 수 있다는 점에서 그 의의가 있다고 본다. 뿐만 아니라 기초간호학에서 근거기반실무의 중요성이 강조되고 있는 시점[5,6]에서 기초간호학적 지식을 비롯한 최상의 근거를 기반으로 임상실무를 수행해야 하는 간호사를 대상으로 근거기반실무역량을 파악하는 것은 향후 간호사가 기초간호학적 연구를 수행하는 연구자로서의 역할과 연구결과를 임상에 적용하는 소비자로서의 역할을 하는데 중요한 시사점을 제공할 수 있을 것으로 본다. 뿐만 아니라 근거기반실무역량 향상은 간호사가 근거기반실무 수행에 있어 기초간호학적 지식의 확장이 요구됨을 파악하는 것과 이를 위한 기초간호학 교육의 필요성을 인식하도록 할 것으로 사료된다. 이러한 점에서 근거기반실무역량에 미치는 영향요인을 규명한 본 연구는 기초간호학 연구 및 교육에 기반자료를 제공할 수 있으리라 사료된다.

## 결론 및 제언

본 연구는 종합병원에 근무하는 간호사의 근거기반실무역량에 미치는 영향을 규명하여 간호사의 근거기반실무역량 강화방안에 필요한 기초자료를 제공하기 위해 시도되었다. 연구 결과 근거기반실무역량에 영향을 주는 요인으로 팔로워십, 그릿 및 연구경험으로 나타났다. 근거기반실무의 확산과 더불어 근거기반실무역량은 간호사에게 필수적으로 요구되고 있다. 이에 추후 간호사의 근거기반실무역량 강화를 위한 전략 마련 시 지시에 효과적으로 따르고 리더의 노력을 지원하며 구조화된 조직을 최대한 활용할 수 있도록 하는 능력인 팔로워십과 장기목표를 위한 끈기와 열정을 의미하는 그릿을 고려하는 것과 연구경험을 증진할 수 있도록 조직 차원의 지원 강화를 제언한다. 특히, 임상문제를 연구로 전환하는 능력이 낮은 것으로 나타났던 점을 비추어 볼 때 연구 관련 교육 프로그램 운영 및 연구 성과에 대한 보상 등 연구를 활성화하는 노력이 근거기반실무 증진에 필요할 것으로 사료된다. 추가적으로 기초간호학적 지식체 확장에 기여하기 위하여 임상에서 근거기반실무에 필요한 기초간호학 지식 요구도를 파악하는 후속 연구를 제언한다.

## Figures and Tables

**Table 1.** General Characteristics of the Participants (N = 203)

Characteristics		n (%)	M ± SD (Min~Max)
Age (yr)	20 ~ 29	84 (41.4)	31.27 ± 5.60 (23~50)
	30 ~ 39	95 (46.8)	
	≥ 40	24 (11.8)	
Sex	Men	13 (6.4)	
	Women	190 (93.6)	
Education level	College	36 (17.7)	
	≥ University	167 (82.3)	
Clinical career (yr)	< 5.0	50 (24.6)	8.83 ± 5.66 (1.16~29.33)
	5.0 ~ 9.9	81 (39.9)	
	10.0 ~ 14.9	44 (21.7)	
	≥ 15.0	28 (13.8)	
Working pattern	Regular day shift	63 (31.0)	
	Shifts	140 (69.0)	
Working department	Emergency room	27 (13.3)	
	General unit	85 (41.9)	
	Intensive care unit	38 (18.7)	
	Integrated nursing care unit	6 (3.0)	
	Outpatient department	23 (11.3)	
	Others^†^	24 (11.8)	
Participation in research-related education	Yes	135 (66.5)	
	No	68 (33.5)	
Membership status or attendance of an academic society	Membership in an academic society	12 (5.9)	
	Attendance of an academic society	85 (41.9)	
	Both	11 (5.4)	
	Neither	95 (46.8)	
Research experience	Yes	108 (53.2)	
	No	95 (46.8)	

M = Mean; SD = Standard deviation; Min = Minimum; Max = Maximum.

† Including nursing education unit, infection control unit, nursing department (physician assistant), not reported.

**Table 2.** Levels of Competency in Evidence-based Practice, Grit, Self-leadership, and Followership (N = 203)

Variables	M ± SD	Min	Max	Range
Competency in evidence-based practice	4.16 ± 0.76	2.00	6.92	1~7
Knowledge/skills	3.99 ± 0.93	1.29	7.00	
Attitudes	4.28 ± 1.10	1.25	6.75	
Practice	4.49 ± 0.83	2.33	7.00	
Grit	2.91 ± 0.30	1.93	3.93	1~4
The passion to become a nursing professional	3.09 ± 0.33	1.60	4.00	
Patient-oriented intrinsic motivation	3.00 ± 0.40	2.00	4.00	
Persistence to achieve long term goals	2.65 ± 0.39	1.60	3.80	
Self-leadership	3.21 ± 0.69	1.11	5.00	1~5
Behavior awareness and volition	3.30 ± 0.77	1.00	5.00	
Constructive cognition	3.24 ± 0.79	1.00	5.00	
Task motivation	3.09 ± 0.83	1.00	5.00	
Followership	2.97 ± 0.54	1.60	4.85	1~5
Independent, critical thinking	2.84 ± 0.53	1.80	4.70	
Active engagement	3.10 ± 0.61	1.10	5.00	

M = Mean; SD = Standard deviation; Min = Minimum; Max = Maximum.

**Table 3.** Differences in Competency in Evidence-based Practice, Grit, Self-leadership and Followership According to General Characteristics (N = 203)

Characteristics	Categories	Competency in Evidence-based Practice		Grit		Self-leadership		Followership	
		M ± SD	t or F or χ^2^ (*p*) Scheffé	M ± SD	t or F or Z or χ^2^ (*p*) Scheffé	M ± SD	t or F or Z or χ^2^ (*p*) Scheffé	M ± SD	t or F or Z or χ^2^ (*p*) Scheffé
Age (yr)	20 ~ 29^a^	4.08 ± 0.71	4.60 (.011)	2.88 ± 0.29	5.65 (.004)	3.22 ± 0.71	4.76 (.010)	2.96 ± 0.63	3.89 (.022)
	30 ~ 39^b^	4.13 ± 0.75	c > b > a	2.88 ± 0.29	c > a,b	3.10 ± 0.64	c > b	2.91 ± 0.42	c > b
	≥ 40^c^	4.59 ± 0.84		3.10 ± 0.34		3.58 ± 0.67		3.25 ± 0.58	
Sex	Men	4.42 ± 0.88	1.26 (.209)	2.93 ± 0.31	−0.31 (.757)^†^	3.13 ± 0.87	−0.11 (.910)^†^	3.21 ± 0.62	−1.54 (.123)^†^
	Women	4.15 ± 0.75		2.91 ± 0.30		3.22 ± 0.68		2.95 ± 0.53	
Education level	College	4.09 ± 0.82	−0.81 (.419)^†^	2.90 ± 0.30	−0.11 (.912)^†^	3.11 ± 0.60	−1.20 (.230)^†^	2.95 ± 0.58	−0.59 (.558)^†^
	≥ University	4.18 ± 0.74		2.91 ± 0.30		3.23 ± 0.71		2.97 ± 0.53	
Clinical career (yr)	< 5.0^a^	4.06 ± 0.77	7.94 (.047)^‡^	2.92 ± 0.32	6.37 (.095)^‡^	3.28 ± 0.71	6.58 (.087)^‡^	2.97 ± 0.59	5.00 (.172)^‡^
	5.0 ~ 9.9^b^	4.14 ± 0.67	d > c > a	2.88 ± 0.24		3.13 ± 0.69		2.92 ± 0.56	
	10.0 ~ 14.9^c^	4.09 ± 0.81		2.84 ± 0.33		3.11 ± 0.65		2.93 ± 0.42	
	≥ 15.0^d^	4.54 ± 0.81		3.07 ± 0.34		3.49 ± 0.66		3.17 ± 0.57	
Working pattern	Regular day shift	4.36 ± 0.75	2.46 (.015)	2.96 ± 0.31	1.84 (.068)	3.28 ± 0.72	0.97 (.335)	3.02 ± 0.52	0.91 (.363)
	Shifts	4.08 ± 0.75		2.88 ± 0.30		3.18 ± 0.68		2.95 ± 0.55	
Working department	Outpatient department	4.21 ± 0.81	9.31 (.097)^‡^	2.92 ± 0.33	6.65 (.248)^‡^	3.11 ± 0.76	2.25 (.813)^‡^	2.99 ± 0.64	2.33 (.801)^‡^
	General unit	4.10 ± 0.71		2.87 ± 0.29		3.22 ± 0.64		2.96 ± 0.57	
	Integrated nursing care unit	3.77 ± 0.84		2.83 ± 0.28		3.39 ± 0.97		3.07 ± 0.77	
	Intensive care unit	4.10 ± 0.86		2.91 ± 0.35		3.11 ± 0.75		2.92 ± 0.50	
	Emergency room	4.17 ± 0.77		2.92 ± 0.28		3.23 ± 0.66		2.99 ± 0.55	
	Others^§^	4.53 ± 0.56		3.00 ± 0.27		3.36 ± 0.67		3.02 ± 0.35	
Participation in research	Yes	4.22 ± 0.76	1.44 (.151)	2.91 ± 0.32	0.53 (.596)	3.22 ± 0.71	0.29 (.775)	2.97 ± 0.53	0.12 (.904)
	No	4.06 ± 0.75		2.89 ± 0.27		3.19 ± 0.65		2.96 ± 0.57	
Membership status or attendance of an academic society	Membership in an academic society^a^	4.36 ± 0.77	7.19 (< .001)	2.91 ± 0.36	33.63 (< .001)^‡^	3.34 ± 0.55	11.28 (.010)^‡^	3.11 ± 0.50	20.75 (<.001)^‡^
			c > b > d		c > a		c > a > d		c > a
	Attendance of an academic society^b^	4.31 ± 0.77		2.98 ± 0.26		3.35 ± 0.67		3.08 ± 0.51	
	Both^c^	4.77 ± 0.54		3.28 ± 0.27		3.42 ± 0.76		3.23 ± 0.48	
	Neither^d^	3.94 ± 0.70		2.80 ± 0.28		3.05 ± 0.68		2.82 ± 0.55	
Research experience	Yes	4.34 ± 0.79	3.57 (< .001)	2.95 ± 0.31	2.34 (.020)	3.31 ± 0.73	2.25 (.026)	3.06 ± 0.56	2.60 (.010)
	No	3.97 ± 0.67		2.85 ±0.28		3.10 ± 0.63		2.87 ± 0.51	

M = Mean; SD = Standard deviation.

^†^Mann-Whitney U test; ^‡^Kruskal-Wallis; ^§^Including nursing education unit, infection control unit, nursing department (physician assistant), not reported.

**Table 4.** Correlations among Competency in Evidence-based Practice, Grit, Self-leadership and Followership (N = 203)

Variables	Grit	Self-leadership	Followership	Competency in evidence-based practice
	r (*p*)			
Self-leadership	.61 (< .001)	1	-	-
Followership	.67 (< .001)	.71 (< .001)	1	-
Competency in evidence-based practice	.62 (< .001)	.54 (< .001)	.72 (< .001)	1

**Table 5.** Factors Influencing Competency in Evidence-based Practice (N = 203)

Variables	B	SE	β	t	*p*	VIF
(Constant)	0.11	0.35		0.32	.746	
Followership	0.74	0.09	0.53	8.29	< .001	1.85
Grit	0.61	0.16	0.24	3.82	< .001	1.84
Research experience^†^	0.17	0.07	0.11	2.27	.024	1.04
F = 83.63, *p* < .001, R^2^ (%) = 55.8, Adjusted R^2^ (%) = 55.1						

SE = Standard error; VIF = Variance inflation factor.

† Dummy coded (reference = research experience “No”).

## Data Availability

Please contact the corresponding author to request data for this study.s
